# A putative *MYB35* ortholog is a candidate for the sex-determining genes in *Asparagus officinalis*

**DOI:** 10.1038/srep41497

**Published:** 2017-02-08

**Authors:** Daisuke Tsugama, Kohei Matsuyama, Mayui Ide, Masato Hayashi, Kaien Fujino, Kiyoshi Masuda

**Affiliations:** 1 Laboratory of Crop Physiology, Research Faculty of Agriculture, Hokkaido University Kita 9 Nishi 9 Kita-ku, Sapporo-shi, Hokkaido 060-8589, Japan

## Abstract

*Asparagus officinalis* (garden asparagus) is a dioecious perennial crop. For agricultural production of *A. officinalis*, male plants have advantages over female plants. The dioecism of *A. officinalis* is determined by the single dominant masculinizing *M* locus, which is involved in tapetal cell development in stamens, but thus far no specific *M* locus genes have been identified. We re-analyzed previously published RNA-Seq data for the *A. officinalis* transcriptome, cloned some genes, and discovered that a putative ortholog of *MYB35*, which is indispensable for tapetal cell development in *Arabidopsis thaliana*, is absent in the genome of female plants in *A. officinalis*. In a reverse transcription-PCR analysis, this gene (*AoMYB35*) exhibited strong expression in stamens in male flowers at an early developmental stage. In an *in situ* hybridization analysis, *AoMYB35* mRNA was detected in tapetal cells in young male flowers. GFP-fused AoMYB35 was detected in the nucleus when expressed in onion epidermal cells. These results suggest that *AoMYB35* is a male-specific gene encoding a putative transcription factor that acts in tapetal cells at an early stage of flower development in *A. officinalis*. Together, the results support the idea that *AoMYB35* is a candidate for one of the *M* locus genes in *A. officinali*s.

Nearly 90% of angiosperms grow bisexual flowers, which have both pistils and stamens^1^. In some of these hermaphroditic plants, pistils and stamens are spatially and/or temporally separated to promote outcrossing, while most of the hermaphroditic plants are self-propagating. The rest (~10%) of the angiosperms grow unisex flowers, which have only pistils or stamens. The unisex flowers with pistils are female flowers, and those with stamens are male. In monoecious plant species, both female and male flowers grow on a single individual. In dioecious species, female flowers grow only on a female individual, and male flowers grow only on a male individual. Dioecy has evolved more than 100 times independently, and 43% of angiosperm families include some dioecious species^2^. Spinach (*Spinacia oleracea*), hop (*Humulus lupulus*), and garden asparagus (*Asparagus officinalis*) are examples of dioecious crops. In date-plum (*Diospyros lotus*), dioecism is determined by two genes, *MeGI* and *OGI*^3^, but it is unclear whether homologs of these genes also regulate dioecism of other species.

*A. officinalis* is a perennial crop, and most of its cultivars are dioecious. In general, male plants are preferred to female plants for agricultural production of *A. officinalis*. This is because the growth rate of male plants is more stable than that of female plants, and because female plants drop seeds, the offspring of which compete for nutrients with older individuals and complicate management of the field. To masculinize seeds and to promote breeding of *A. officinalis*, supermale plants, which generate only male offspring, are utilized. Supermale plants can be obtained by either anther culture or selfing a hermaphroditic individual^4^,^5^.

The chromosomes of female *A. officinalis* plants are morphologically indistinguishable from those of male plants^6^. The dioecism of *A. officinalis* is thought to be determined by the single dominant masculinization-promoting *M* locus. Because the *M* locus does not affect the morphology of the chromosomes, it is thought to be a relatively small region, and to contain a small number of genes^6^,^7^. The genotype of female plants is *mm*, that of male plants is *Mm*, and that of supermale plants is *MM*. A male-specific DNA marker linked to the *M* locus has been developed^8^,^9^, but no specific *M* locus genes have been identified. In male flowers of *A. officinalis*, pistils emerge but stop developing. In female flowers, stamens emerge, but the anthers start degenerating immediately before tapetal cells, which surround and mature microspores, develop to complete meiosis^10^. In a previous RNA-Seq analysis using *A. officinalis*, genes possibly regulating the maturation of microspores were more strongly expressed in male flowers than in female flowers, while genes possibly regulating either tapetal development or the early stages of anther development were not expressed^11^. These findings suggest that *M* locus genes regulate tapetal development and/or the early stages of microspore maturation.

Here, we show that a putative ortholog of *MYB35*, which is indispensable for tapetal development in the model plant *Arabidopsis thaliana*^12^, is a strong candidate for one of the *M* locus genes in *A. officinalis*.

## Results and Discussion

### The putative *MYB35* ortholog is absent in the genome of female *A. officinalis* plants

**Figure 1:**
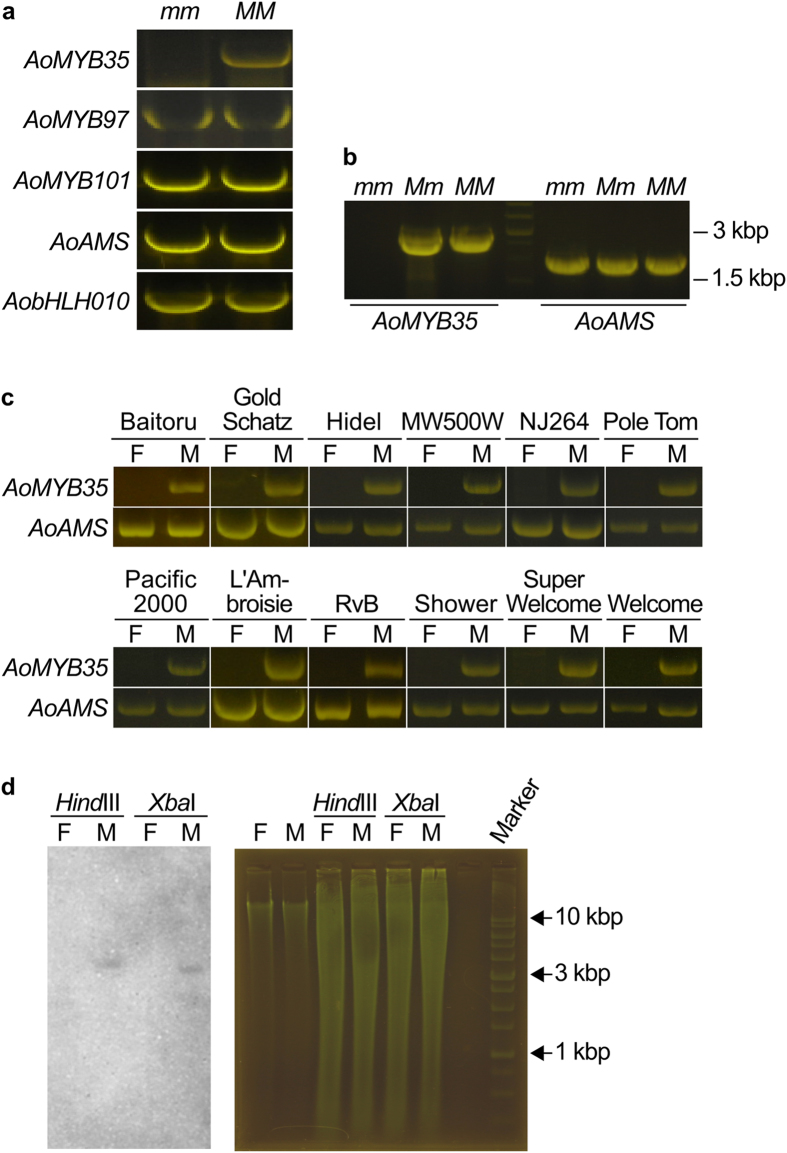
*AoMYB35* is absent in female *Asparagus officinalis* plants. (**a**) Genomic PCR analysis of the genes encoding MYB and bHLH transcription factors that could regulate anther development. Genomic DNA was extracted from female (*mm*) and supermale (*MM*) plants of the *A. officinalis* cultivar Gijnlim, and used as the PCR template. (**b**) Genomic DNA of male (*Mm*) plants of Gijnlim was also subjected to the PCR analysis of *AoMYB35* and *AoAMS* as in panel a. The middle (fourth) lane shows the pattern of a DNA size marker. (**c**) Genomic DNA was prepared from female (F) and male (M) plants of the indicated cultivars (MW500W: Mary Washington 500 W; NJ264: New Jersey 264; RvB: Ruhm von Braunschweig), and subjected to the PCR analysis of *AoMYB35* and *AoAMS* as in panel a. Experiments were repeated more than three times for each gene in the panels a–c, and representative cropped gel images are shown. (**d**) Southern blot analysis of *AoMYB35*. Twenty μg genomic DNA of female (F) and male (M) plants of NJ264 was digested by either *Xba*I or *Hind*III, and subjected to Southern blotting. Signals were detected using a digoxigenin (DIG)-labeled *AoMYB35*-specific probe (left panel). In the right panel, gel images are shown as loading controls (Ctrl: 1.5 μg undigested DNA was run in each lane; *Hind*III and *Xba*I: 20 μg digested DNA was run in each lane). Experiments were repeated three times, and a representative result is shown.

An RNA-Seq analysis of the *A. officinalis* transcriptome^11^ was unable to find homologs of *AtMYB35* and *AtMYB33*, which regulate tapetal development in Arabidopsis^12^,^13^. However, we re-analyzed the same RNA-Seq data and identified close homologs of these genes. The FPKM (fragments per kilobase of exon per million mapped fragments) values obtained by the re-analysis of the RNA-Seq data suggest that these genes and other genes encoding MYB and basic helix-loop-helix (bHLH) transcription factors that could regulate tapetal functions (see ref. 14 for a review) are expressed more strongly in male flowers than in female flowers in *A. officinalis* (Supplementary Fig. S1 and Supplementary Table S1). Reverse transcription (RT)-PCR and genomic PCR were performed to confirm the sequences of these genes. In these analyses, most of the genes such as *AoAMS*, a close Arabidopsis homolog of which (*AtAMS*) regulates microspore maturation downstream of *AtMYB35*^12^,^15^, could be amplified even when female-derived DNA was used as the template. However, interestingly, the fragments of the close *MYB35* homolog in *A. officinalis (AoMYB35*) could be amplified only when male- or supermale-derived DNA was used as the template, and not when female-derived DNA was used (Fig. 1a–c, Supplementary Fig. S2 and S3). These results raise the possibility that *AoMYB35* is absent in the genome of female *A. officinalis* plants.

Aspof_comp61397_c0_seq6 is a contig that was generated in the previous RNA-Seq analysis^11^, and corresponds to *AoMYB35* (Supplementary Table S1). The 5′ end of *AoMYB35* could not be obtained in our 5′ RACE (rapid amplification of cDNA ends) experiment, but a 3′ RACE analysis and an RT-PCR analysis with various primers support the idea that Aspof_comp61397_c0_seq6 corresponds to the putative full-length cDNA of *AoMYB35* (Supplementary Fig. S4). In Arabidopsis, AtMYB80 (also known as MS188 or AtMYB103), which is indispensable for microspore maturation^16^, and AtMYB35 are the closest homologs of each other. The deduced amino acid sequence of AoMYB35 was more similar to the sequence of AtMYB35 than AtMYB80 (Supplementary Fig. S5), supporting the idea that *AoMYB35* is a putative *MYB35* ortholog in *A. officinalis*. The genomic region corresponding to *AoMYB35* is expected to be 2,726 bases long with three introns (Supplementary Fig. S6).

In genomic PCR with various *AoMYB35*-specific primer pairs, signals were not obtained with any of them when female-derived DNA was used as the template (Supplementary Fig. S7). In Southern blotting, *AoMYB35* signals were detected as single bands on male-derived DNA, but not on female-derived DNA (Fig. 1d). These results further support the idea that *AoMYB35* is a male-specific gene in *A. officinalis*.

### *AoMYB35* is expressed at an early stage of anther development in male flowers, and its product is localized to the nucleus

**Figure 2:**
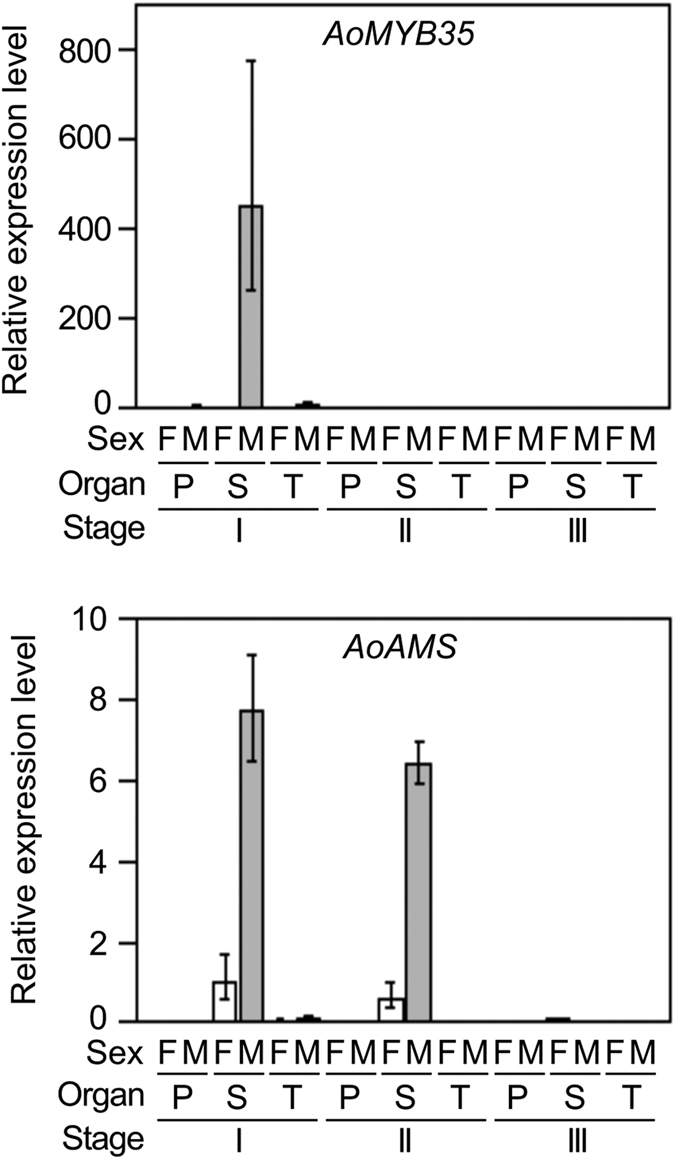
*AoMYB35* is expressed specifically in stamens at the early stage of male flower development in *Asparagus officinalis*. Young female (F) and male (M) flower buds were classified into the developmental stages I (early) to III (late) on the basis of their sizes. RNA was extracted from pistils (P), stamens (S), and tepals (T) of these flower buds, and used for cDNA synthesis. Relative expression levels were calculated using the comparative cycle threshold method with the gene encoding ubiquitin-ribosomal peptides fusion protein as the internal control. *AoAMS* is a possible downstream target gene of AoMYB35, and its expression level is shown as a control. Values are presented as means ± SD of three biological replicates.

**Figure 3:**
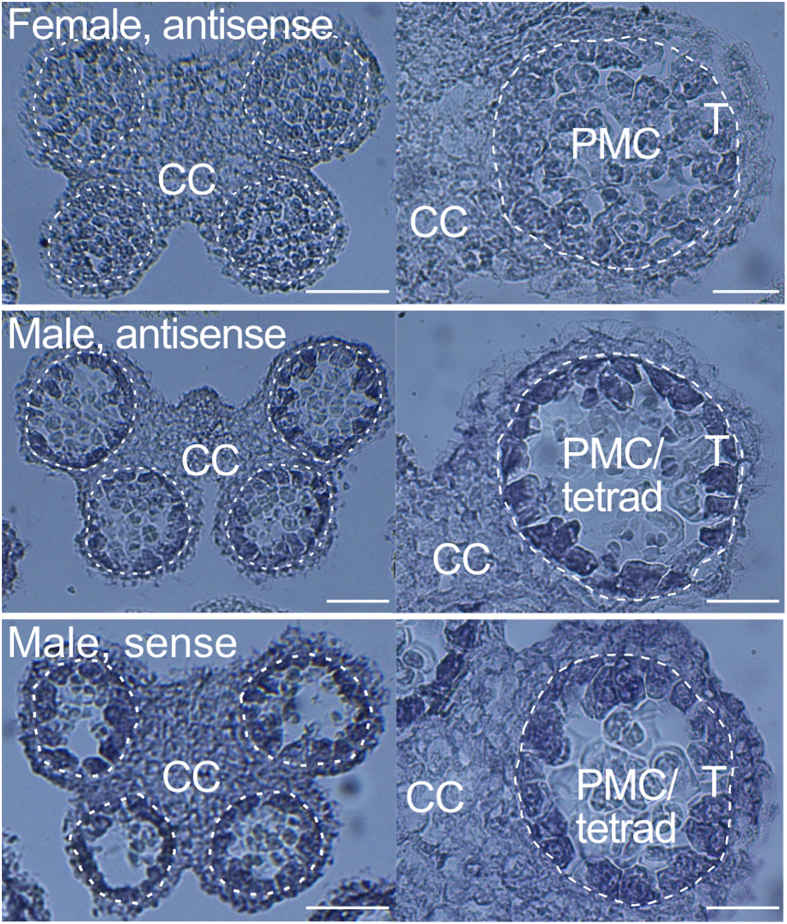
*AoMYB35* is expressed in tapetal cells in male flowers of *Asparagus officinalis*. Young flower buds of female and male flowers of the *A. officinalis* cultivar NJ264 were sampled at the meiotic stage, thin-sectioned, and subjected to *in situ* hybridization using the sense and antisense probes to detect *AoMYB35* mRNA (combinations of the sex and the probes are indicated). Experiments were repeated more than three times, and representative images of anthers are shown. Broken lines indicate peripheries of the tapetum. T, PMC/tetrad and CC indicate the positions of tapetal cells, meiotic pollen mother cells and connective cells, respectively. Note that in the male samples, connective cells and other cells surrounding the tapetum were more strongly stained when the sense probe was used than when the antisense probe was used. Bars = 100 μm for left panels, and 50 μm for right panels.

In the RNA-Seq analysis, the FPKM value of *AoMYB35* in male flowers was higher at the premeiotic stage than at the meiotic or postmeiotic stage (Supplementary Table S1). The expression of *AoMYB35* in pistils, stamens, and tepals in young female and male flowers at different developmental stages (see Supplementary Fig. S8) was examined by quantitative RT-PCR. In agreement with the above result of the RNA-Seq analysis, *AoMYB35* expression was high in stamens in male flowers at the developmental stage I, which should correspond to the premeiotic tapetal cell-developing stage, and low in pistils and tepals in male flowers at the same stage. Hardly any *AoMYB35* expression was detected in the other samples studied (Fig. 2, upper panel). In a previous study, in Arabidopsis, the expression of *AtAMS* in flowers was weaker in the *AtMYB35*-deficient mutant (*atmyb35*) than in the wild type^12^. In quantitative RT-PCR, the expression level of *AoAMS* was high in stamens in male flowers at the stage I and the stage II, which corresponds to an early postmeiotic stage. A lower yet fair level of *AoAMS* expression was detected in stamens in female flowers at the stages I and II. Hardly any *AoAMS* expression was detected in the other samples studied (Fig. 2, lower panel). These results support the idea that *AoAMS* is a possible downstream target gene of AoMYB35. In an *in situ* hybridization analysis, when an antisense probe was used to detect *AoMYB35* mRNA, signals were detected in tapetal cells in male flowers, but not in female flowers (Fig. 3, top and middle panels). When a sense probe was used, the pattern of signals was somewhat similar to that obtained with the antisense probe, but seemed less specific to tapetal cells (Fig. 3, lower panel). These results suggest that *AoMYB35* is expressed in tapetal cells at the early stage of male flower development.

**Figure 4:**
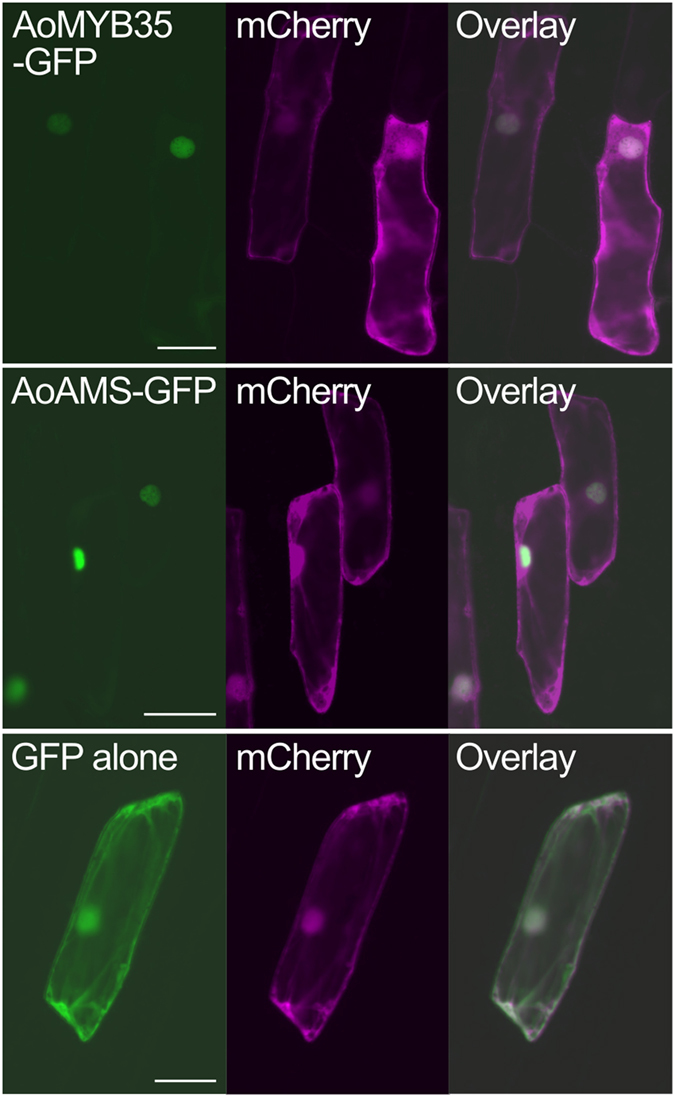
AoMYB35 is localized to the nucleus. GFP, GFP-fused AoMYB35 (AoMYB35-GFP), or GFP-fused AoAMS (AoAMS-GFP) was co-expressed with mCherry in onion epidermal cells, and their signals were observed by fluorescence microscopy. More than 10 transformed cells were observed for each combination of constructs, and representative results are shown. Bars = 100 μm.

When AoMYB35 was expressed as a GFP-fused protein in onion epidermal cells, its signals were detected in the nucleus (Fig. 4), supporting the idea that AoMYB35 is localized to the nucleus to act as a transcription factor.

### Not only *AoMYB35* but also other genes may be present in the *M* locus

In flowers of wild-type Arabidopsis plants and male *A. officinalis* plants, callose is deposited around PMCs when they undergo meiosis to differentiate into pollen tetrads. The deposited callose is degraded by the callase enzymes that are secreted by tapetal cells when tetrads mature into pollen grains. In flowers of *atmyb35*, callose is deposited around PMCs, but the deposited callose remains undegraded^10^,^12^. States of DNA and callose deposition in and around PMCs of *A. officinalis* were re-examined. In both female and male flowers of *A. officinalis*, DNA of PMCs was detected as dots (Supplementary Fig. S9a), suggesting that their DNA is condensed. The DNA condensation in PMCs in female flowers might occur as the first step for meiosis as in male flowers, although it might be a sign of another biological process such as cell death. In agreement with a previous report^10^, only a small amount of callose was detected around PMCs in female flowers (Supplementary Fig. S9b, stages i and ii). The callose accumulated around PMCs in female flowers seemed to remain undegraded until a later stage at which tapetal cells started degenerating (Supplementary Fig. S9b, stage iii). Deformation of PMCs follows the degeneration of tapetal cells in female flowers, and at this stage, no callose was detectable (Supplementary Fig. S9b, stage iv). The callose deposition patterns between *atmyb35* and female *A. officinalis* plants would not be consistent. This inconsistency does not completely exclude the possibility that *AoMYB35* regulates the callose deposition around PMCs, but it may also be possible that an *M* locus gene other than *AoMYB35* regulates the callose deposition. *AtCDM1* is a zinc-finger transcription factor required for the callose deposition around the PMCs in Arabidopsis^17^. Among the contigs generated by the previous RNA-Seq analysis^11^, two contigs, Aspof_comp52054_c0_seq1 and Aspof_comp59805_c1_seq1, have high similarity to *AtCDM1*. On the basis of the FPKM values, the corresponding *A. officinalis AtCDM1*-like genes (*AoCDML1* and *AoCDML2*) are more weakly expressed in female flowers than in male flowers at the premeiotic stage (Supplementary Table S1). The defects in callose deposition around PMCs in *A. officinalis* female flowers might be due to this weak expression of *AoCDML1* and *AoCDML2*. In genomic PCR, both *AoCDML1* and *AoCDML2* were detected either when female-derived DNA was used as the template or when male-derived DNA was used (Supplementary Fig. S10), suggesting that female *A. officinalis* plants as well as male have these genes. A gene regulating the expression of *AoCDML1* and *AoCDML2* is likely to be another candidate for the *M* locus genes. However, neither such a gene nor a direct regulator of the expression of *AtCDM1* has been identified thus far.

In male *A. officinalis* flowers, pistils stop growing. This process is likely to be under the control of not *AoMYB35* but another *M* locus gene because *AoMYB35* is hardly expressed in pistils (Figs 2 and 3). Further studies are needed to identify all the *M* locus genes in *A. officinalis*.

## Methods

### Analysis of RNA-Seq data

The read data for RNA-Seq with *A. officinalis*^11^ were downloaded from the Sequence Read Archive (SRA) of The National Center for Biotechnology Information (NCBI) (see Supplementary Table S2 for accession numbers). The contigs generated by these reads in a previous study^11^ were downloaded from Dryad (http://dx.doi.org/10.5061/dryad.92c60)^18^. The 3′ ends of reads with <20 Phred scores were trimmed with the FASTX toolkit (http://hannonlab.cshl.edu/fastx_toolkit/) according to the previous study^11^. The trimmed reads were mapped to the contigs using Bowtie2 version 2.2.6 (http://bowtie-bio.sourceforge.net/bowtie2/index.shtml)^19^ with the option allowing 1-base mismatch. This mapped approximately 90% of the reads to the contigs in each data set. The mapped reads were counted using featureCounts (http://bioinf.wehi.edu.au/featureCounts/)^20^. To identify Arabidopsis homologs for the *A. officinalis* genes, the BLASTN program in the NCBI BLAST+ suite^21^ was run using the above contigs as queries and the Arabidopsis cDNA sequence set, which was downloaded from The Arabidopsis Information Resource (TAIR), as the database. The count data and other relevant data were managed with SQLite version 3.9 (www.sqlite.org) to obtain FPKM values and to extract contigs of interest. The genes and corresponding contigs analyzed in this study are shown in Supplementary Table S1.

### RT-PCR and genomic PCR

For RT-PCR to clone the *AoMYB35* cDNA, total RNA was extracted using TRIzol reagent (Thermo Fischer Scientific, Waltham, MA) from young flower buds of female and male plants of the *A. officinalis* cultivar New Jersey 264 (NJ264) maintained for 10–20 years in the open field of Hokkaido University. First-strand cDNA was synthesized from 2 μg total RNA using the M-MLV reverse transcriptase (Promega, Fitchburg, WI) and the primer 5′-AAGCAGTGGTAACAACGCAGAG(T)_30_VN-3′. The cDNA solution was diluted 20 times with distilled water, and used as the PCR template. The *MYB35* cDNA fragments were amplified by PCR using this template, the primers shown in Supplementary Table S3, and the KOD FX Neo DNA polymerase (Toyobo, Osaka, Japan). The PCR products were cloned into the *Sma*I site of pBluescript II SK^−^ (Agilent Technologies, Santa Clara, CA), generating pBS-AoMYB35c, and sequenced with the 3130 Genetic Analyzer (Applied Biosystems, Foster City, CA). This confirmed that the contig Aspof_comp61397_c0_seq6, which was generated in a previous study^11^, should correspond to the *AoMYB35* cDNA. Primers for the other genes amplified by RT-PCR are shown in Supplementary Table S3. The deduced amino acid sequence of AoMYB35 was aligned with the amino acid sequences of AtMYB35 (AT3G28470) and AtMYB80 (AT5G56110) using Clustal W^22^.

To clone the genomic region corresponding to *AoMYB35*, genomic DNA was extracted using the DNeasy Plant Mini kit (Qiagen, Tokyo, Japan) from cladodes (pseudo-leaves) of female, male, and supermale plants of the *A. officinalis* cultivar Gijnlim maintained for 10–20 years in the open field of Hokkaido University. The *AoMYB35* genomic fragments, which correspond to its coding sequence (CDS), 5′ and 3′ untranslated regions, and introns were amplified by PCR using the genomic DNA solution as the template, primers shown in Supplementary Table S3, and the PrimeStar GXL DNA polymerase (Takara Bio, Shiga, Japan). To isolate the promoter region of *AoMYB35*, TAIL (thermal asymmetric interlaced)-PCR was performed as previously described^23^ using the genomic DNA solution as the template and the primers shown in Supplementary Table S3. The PCR products were cloned into the *Sma*I site of pBluescript II SK^−^, and sequenced as described above. The GenBank accession number for the sequence of the genomic region of *AoMYB35* is KX684199. The putative TATA box of *AoMYB35* was predicted using the GPMiner program (http://gpminer.mbc.nctu.edu.tw/index.php)^24^. Primers for the other genes amplified by genomic PCR are shown in Supplementary Table S3.

For the genomic PCR with various cultivars, genomic DNA was extracted using DNeasy Plant Mini from cladodes of female and male plants of the cultivars NJ264, Gold Schatz, L’Ambroisie, and Ruhm von Braunschweig (RvB), and male plants of the cultivars KBFX3-9, J. Deluxe, Guelph, Zuiyou, Grande, Green Tip, and Grune Krone maintained for 10–20 years in the open field of Hokkaido University. Genomic DNA of female and male plants of the cultivars Baitoru, Hidel, Mary Washington 500 W (MW500W), Pole Tom, Pacific 2000, Shower, Super Welcome, and Welcome, and male plants of the cultivars Green Fit, Santaclaus, Zenyu Jodel, Burgundy, High Catch, and Manmimurasaki were kindly provided by Dr. Yuichi Uno (Kobe University, Japan). PCR was run using the genomic DNA from these cultivars as the template, primers shown in Supplementary Table S3, and the PrimeStar GXL DNA polymerase.

Signals of the PCR products in agarose gels were visualized with the Safe Imager blue light transilluminator (Thermo Fischer Scientific) and either Atlas ClearSight (Bioatlas, Tartu, Estonia) or RedSafe Nucleic Acid Staining Solution (Intron Biotechnology, Seongnam, Korea), and photographed with the PowerShot A630 digital camera (Canon, Tokyo, Japan). Gel images were processed with the GIMP (http://www.gimp.org/) and Inkscape (http://www.inkscape.org) programs.

For quantitative RT-PCR, the young flower buds of female plants of the *A. officinalis* cultivar Atlas, which were maintained in the open field of Hokkaido University, and those of male plants of the cultivar NJ264 were classified into the developmental stages I (early) to III (late) on the basis of growth of pistils and stamens (see Supplementary Fig. S8). Pistils, stamens, and tepals in these flower buds were separated using a small, finely sharpened hand-made knife. Total RNA was extracted from these samples using the Plant RNA Isolation Mini kit (Agilent Technologies), and cDNA was synthesized from 1 μg of the total RNA using the ReverTra Ace qPCR RT Master Mix with gDNA Remover kit (Toyobo). The cDNA solution was diluted 10 times with distilled water, and used as the PCR template. The quantitative RT-PCR was run using this template, the primers shown in Supplementary Table S3, GoTaq qPCR Master Mix (Promega), and the CFX Connect real-time PCR detection system (Bio-Rad, Hercules, CA). Relative expression levels were calculated using the comparative cycle threshold method with the gene encoding ubiquitin-ribosomal peptides fusion protein (GenBank accession number: X66875.1) as the internal control.

### Southern blotting

The 3′ region of *AoMYB35* was amplified by PCR using pBS-AoMYB35g as the template, the PrimeStar GXL DNA polymerase, the DIG DNA labeling mix (Roche Diagnostics, Basel Switzerland), and the following primer pair: 5′-CAGCCACAAGCACCACATTGGATG-3′ and 5′-GGCAATAACTAGATGACATAATTAGC-3′. The PCR products were gel-purified, and the resulting solution was used as the probe solution. Genomic DNA was prepared from young flower buds of female and male plants of NJ264 using the DNeasy Plant Mini kit. Twenty μg genomic DNA was digested by either *Hind*III or *Xba*I for 4 h at 37 °C, and electrophoresed on a 0.8% (w/v) agarose gel in 0.5× Tris-acetate/EDTA running buffer. The gel was incubated in a denaturation solution (1.5 M NaCl and 0.5 M NaOH) for 15 min at room temperature twice, in a neutralization solution (1.5 M NaCl and 0.5 M Tris-HCl, pH 7.5) for 15 min at room temperature twice, and in 10× SSC (1× SSC: 0.15 M NaCl, 0.015 M sodium citrate, pH 7.0) for 15 min at room temperature once. The DNA in the gel was then capillary-transferred to the Biodyne B nylon membrane (Pall, Port Washington, NY). The membrane was incubated in DIG Easy Hyb (Roche Diagnostics) at 42 °C for 1 h, and then in a hybridization solution (the probe solution diluted 2,000 times with DIG Easy Hyb) at 42 °C for 8 h. The membrane was washed twice with a primary wash solution (0.1% (w/v) sodium dodecyl sulfate in 2× SSC) for 5 min at room temperature, and twice with a secondary wash solution (0.1% (w/v) sodium dodecyl sulfate in 0.5× SSC) for 20 min at 65 °C. The membrane was then briefly washed with TBST (Tris-buffered saline with Tween 20: 0.15 M NaCl, 20 mM Tris-HCl, pH 7.4, 0.2% (v/v) Tween 20), incubated in a blocking solution (TBST containing 3% (w/v) skim milk) at room temperature for 30 min, and in an antibody solution (Anti-Digoxigenin-AP, Fab fragments (Roche Diagnostics) diluted 10,000 times with the blocking solution) at room temperature for 1 h. The membrane was washed four times with TBST for 5 min at room temperature, and signals were detected using the CDP-Star, ready-to-use solution (Roche Diagnostics) and the LumiVision Pro imager (Aisin Seiki, Aichi, Japan). Gel images were obtained as described in the “RT-PCR and genomic PCR” subsection. Images were processed using GIMP and Inkscape.

### *In situ* hybridization

The 3′ region of *AoMYB35* cDNA was amplified by PCR using pBS-AoMYB35c as the template and one of the following primer pairs: (1) 5′-TAATACGACTCACTATAGGGCAGCCACAAGCACCACATTGGATG-3′ (T7 promoter sequence is underlined) and 5′-GGCAATAACTAGATGACATAATTAGC-3′ (for the sense probe); (2) 5′-CAGCCACAAGCACCACATTGGATG-3′ and 5′- TAATACGACTCACTATAGGGCAATAACTAGATGACATAATTAGC-3′ (T7 promoter sequence is underlined) (for the antisense probe). The PCR products were gel-purified, and used as the template to synthesize the DIG-labeled RNA probes with DIG RNA Labeling Kit (SP6/T7) (Roche Diagnostics).

Young female and male flower buds of the *A. officinalis* cultivar NJ264 were sampled at the meiotic stage, fixed in the FAA solution (4% (w/v) formaldehyde, 5% (v/v) acetic acid, 50% (v/v) ethanol) at room temperature for 16 h. The samples were then dehydrated, embedded in paraffin, thin-sectioned, fixed on glass slides, deparaffinized, treated with 0.1 μg/ml proteinase K for 30 min at room temperature, re-fixed in 4% (w/v) formaldehyde for 10 min at room temperature, acetylated, and dried for hybridization essentially as described previously^25^. The RNA probe described above was diluted 150 times with the hybridization buffer (50% (v/v) formamide, 10% (w/v) dextran sulfate, 0.02% (w/v) bovine serum albumin, 0.02% (w/v) polyvinyl pyrrolidone, 0.1% (w/v) yeast RNA, 0.3 M NaCl, 10 mM 2-morpholinoethanesulfonic acid, pH 5.8), and hybridization was performed overnight at 45 °C in the hybridization buffer with the probe. Samples were incubated in 2× SSC at room temperature for 5 min twice, and in 0.2× SSC for 1 h at 60 °C twice for washing. Samples were then reacted at room temperature for 2 h with the Anti-Digoxigenin-AP, Fab fragments diluted 5,000 times in TBST, washed three times with TBST, equilibrated with the pre-detection buffer (0.1 M NaCl, 0.1 M Tris-HCl, pH 9.5), and incubated at room temperature in the solution containing the NBT/BCIP Ready-to-Use tablets (Roche Diagnostics) until signals became visible (approximately 1 h). Samples were then washed briefly with distilled water, and immediately observed with an optical microscope equipped with the LUMIX DMC-G6 digital camera (Panasonic, Osaka, Japan). Images were processed with GIMP and Inkscape.

### Expression of GFP-fused proteins

The CDSs of *AoMYB35* and *AoAMS* were obtained by RT-PCR as described above (see Supplementary Table S3 for the primers used). The *AoMYB35* CDS fragment was digested by *Xba*I and *Spe*I, and cloned into the *Xba*I-*Spe*I site of the pBS-35SMCS-GFP vector^26^. The *AoAMS* CDS fragment was digested by *Xba*I and *Sal*I, and cloned into the *Xba*I-*Sal*I site of pBS-35SMCS-GFP. One of these constructs and pBS-35SMCS-mCherry^27^ (0.5 μg each) were mixed, and co-introduced into onion (*Allium cepa*) epidermal cells using the Biolistic PDS-1000/He particle delivery system (Bio-Rad). Cells were incubated for 24 h at room temperature after being transformed, and signals were observed using the BX50 epifluorescence microscope (Olympus, Tokyo, Japan) equipped with the ORCA-ER-1394 digital camera (Hamamatsu Photonics, Hamamatsu, Japan). The fluorescence mirror units U-MGFPHQ and U-MWIG2 (Olympus) were used to image GFP and mCherry, respectively. Images were processed with GIMP and Inkscape.

### Analyses of callose and DNA in histological sections

Young flower buds of the cultivar RvB were embedded in paraffin, semi-thin-sectioned, and rehydrated in distilled water as described previously^25^. To detect callose, the section was rinsed with a 70 mM phosphate buffer (pH 7.0), stained with 0.1% (w/v) aniline blue in the phosphate buffer, and then washed with the phosphate buffer. To detect DNA, the section was subjected to Feulgen stain^28^. The section was hydrolyzed in 5 N HCl at room temperature for 30 min, washed with distilled water, and then incubated in Shiff’s reagent (Merck Millipore, Billerica, MA) at room temperature for 1 h, and washed with a sulfurous acid solution (Wako Pure Chemical Industries, Osaka, Japan). Signals of aniline blue were detected with the epifluorescence microscopy as described in the “Expression of GFP-fused proteins” subsection. The fluorescence mirror unit U-MWU2 (Olympus) was used. Signals of basic fuchsin in the Shiff’s reagent were detected with the TCS SP5 confocal laser scanning microscope (Leica Microsystems, Wetzlar, Germany) using a HeNe laser. Images were processed with the Canvas X software (ACD Systems, Victoria, Canada).

## Additional Information

**How to cite this article:** Tsugama, D. *et al*. A putative *MYB35* ortholog is a candidate for the sex-determining genes in *Asparagus officinalis. Sci. Rep.* **7**, 41497; doi: 10.1038/srep41497 (2017).

**Publisher's note:** Springer Nature remains neutral with regard to jurisdictional claims in published maps and institutional affiliations.

## Supplementary Material

Supplementary Figures

Supplementary Table S1

Supplementary Table S2

Supplementary Table S3
