# Supplementary material for: A putative MYB35 ortholog is a candidate for the sex-determining genes in Asparagus officinalis
Source: Sci Rep. 2017 Feb 8;7:41497. doi: 10.1038/srep41497 (PMC5296871; doi:10.1038/srep41497)
Supplement: Supplementary Figures [file srep41497-s1.pdf]

Supplementary figures for:

A putative *MYB35* ortholog is a candidate for the sex-determining genes in *Asparagus officinalis*

Daisuke Tsugama, Kohei Matsuyama, Mayui Ide, Masato Hayashi, Kaien Fujino, and Kiyoshi Masuda*

Laboratory of Crop Physiology, Research Faculty of Agriculture, Hokkaido University

Kita 9 Nishi 9 Kita-ku, Sapporo-shi, Hokkaido 060-8589, Japan

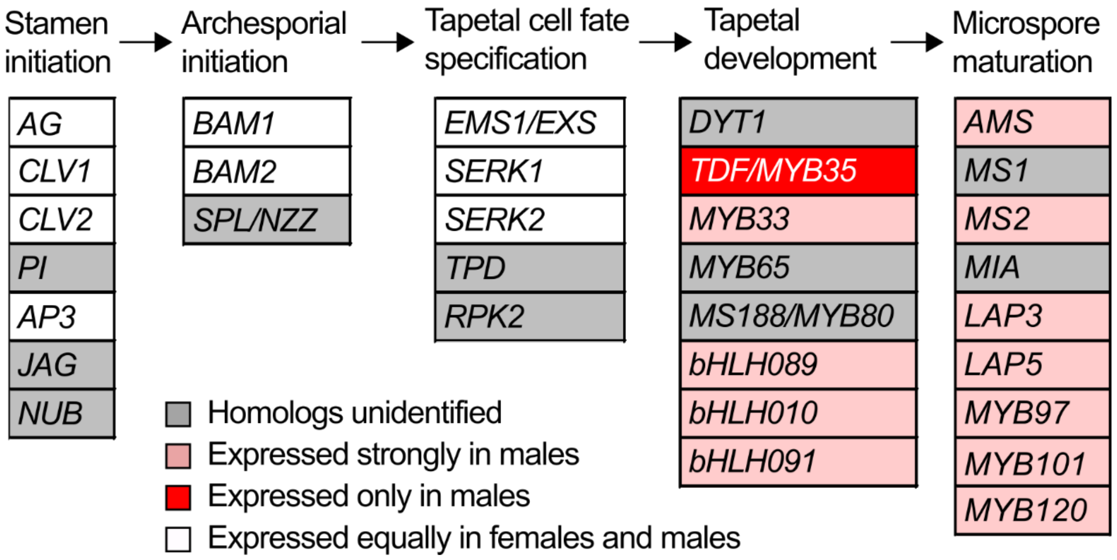

Figure S1. Genes regulating stamen development in *Arabidopsis thaliana* and the expression of their homologs in *Asparagus officinalis*. The figure is a modified version of a figure in a previous study[11]. For the genes indicated by grey, no close homologs have been identified in *Asparagus officinalis*. For the genes indicated by pale red, their homologs in *A. officinalis* were more highly expressed in male flowers than in female flowers. The expression of the close homolog of *MYB35* in *A. officinalis* was detected in male flowers but not at all in female, as indicated by red. For the *A. officinalis* homologs of the genes indicated in white, no significant difference was observed in their expression levels between female and male flowers. The expression levels of those genes are based on the FPKM values obtained in the re-analysis of RNA-Seq data.

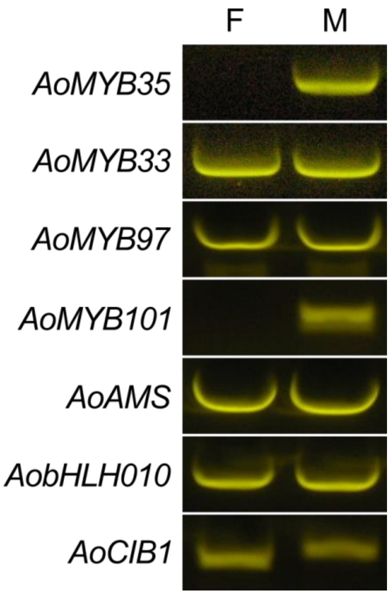

Figure S2. *AoMYB35* transcripts are absent in female flowers of *Asparagus officinalis*. RNA was extracted from young female (F) and male (M) flowers of the *A. officinalis* cultivar NJ264, and used for cDNA synthesis for the RT-PCR analysis of transcripts of the indicated genes. Experiments were repeated three times for each gene, and representative results are shown.

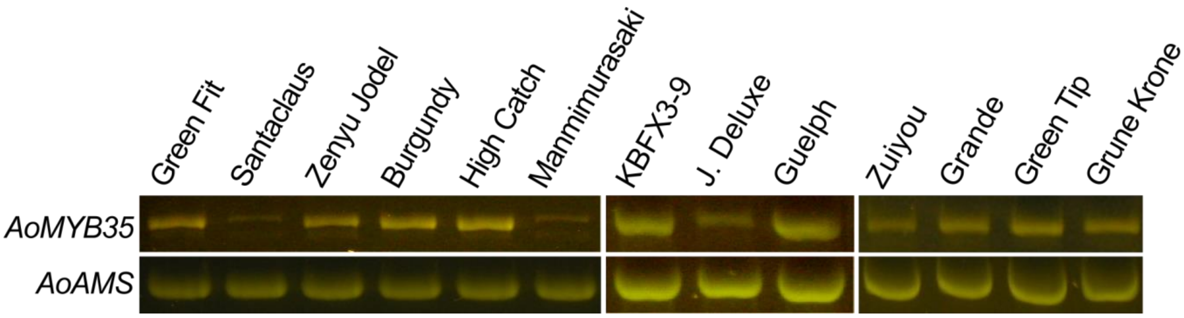

Figure S3. *AoMYB35* is present in the genome of male plants of various cultivars of *Asparagus officinalis*. The genomic DNA was prepared from male plants of the indicated cultivars, and used as the template for the PCR analysis of *AoMYB35* and *AoAMS*. Experiments were performed more than three times for each template, and representative results are shown.

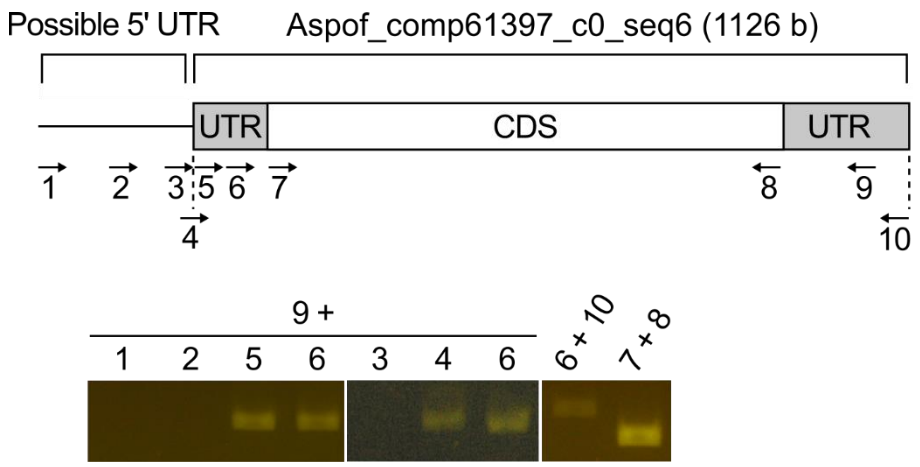

Figure S4. Aspof_comp61397_c0_seq6 is likely to correspond to the full-length mRNA of *AoMYB35*. Aspof_comp61397_c0_seq6 is a contig that was generated in a previous RNA-Seq analysis[11] and corresponds to *AoMYB35* (putative CDS and untranslated regions (UTRs) are shown). The 5’ flanking sequence of the genomic region of *AoMYB35* was obtained by TAIL-PCR, and used as the possible 5’ UTR sequence for the *AoMYB35* mRNA. RNA was extracted from young male flowers of the *Asparagus officinalis* cultivar NJ264, and used for cDNA synthesis. RT-PCR was performed using this cDNA as the template and the primers 1-10 (their annealing sites and combinations are shown). The sequence of the 3’ end of *AoMYB35* cDNA was independently determined by 3’ RACE, and it was exactly the same as the sequence of one end of Aspof_comp61397_c0_seq6. Experiments were performed three times for each primer combination, and representative results are shown.

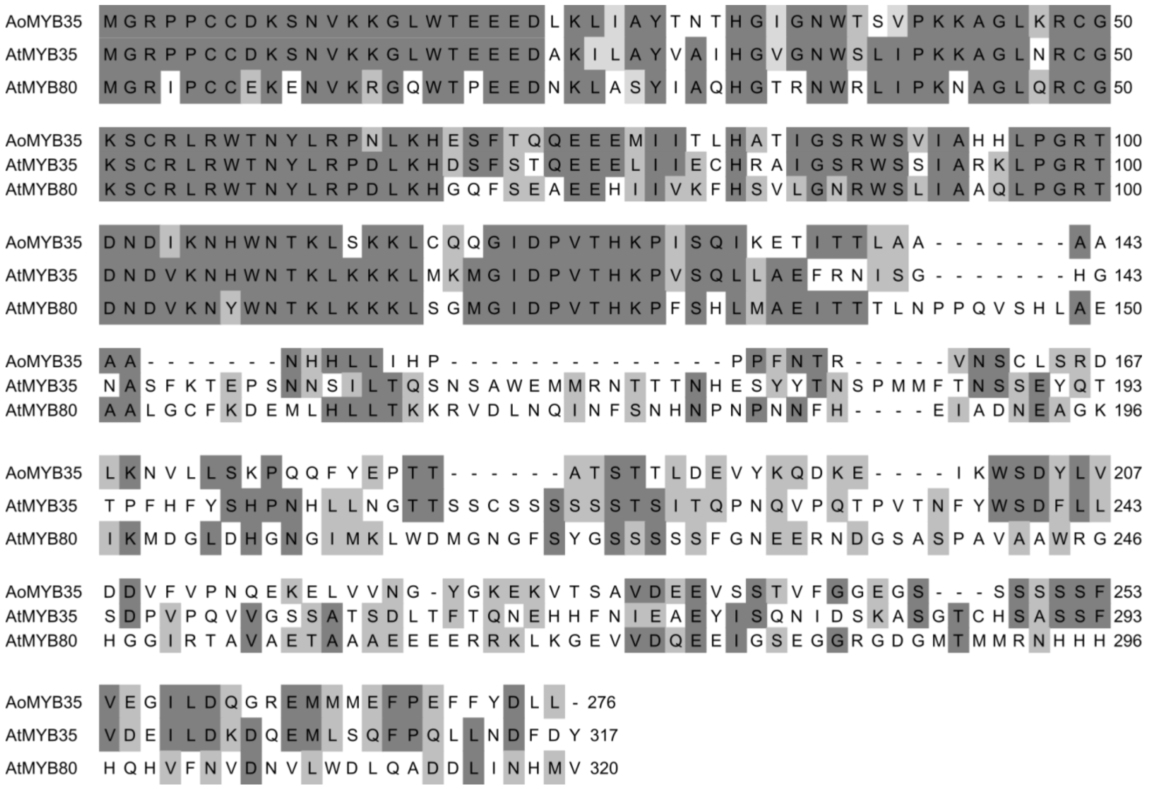

Figure S5. Alignment of the amino acid sequences of AoMYB35, AtMYB35, and AtMYB80.

GAACCTGATTGGCACTTGTGAGCCAAGTAATTGAGGTGTGGGTGCGGTTTGCAGCCCGGAAACCACAAACT
TAATTTAAAATTTTCACATCATATCTCAAACATTAACCGTTTTTTGAACATAAATCATGCATGCCATCATT
TGATAAATGAGAGTGAATCTCATGCTTTTCTTGCCTATCCTTCTTAAAAAAGCTTTTAAATACATCTACTT
CTAACTCTGTGCATAATTGGTTTCATCATCATTGTACCTCAGCCCTTAAGGTTAAATGTCGCATCACCATG
GCATCTCTTATTCTCTCTTTTACCAAAAAGAATTTACATTAACTTATAGCCTAATTATAACTTGGTCATTT
TTTATATTTTATGATTTGAAAAGTTTTATGTTAATATTTTGTAGAATTAGGAGATGGGCAGGCCTCCATGC
TGCGATAAATCCAACGTGAAGAAGGGACTTTGGACTGAGGAAGAAGATTTGAAGCTAATAGCTTATACCAA
CACTCATGGAATAGGAAATTGGACTTCTGTTCCAAAGAAAGCAGGTTCTTTTTACGTAGCTAATTGGTTGA
TTTTCTTCAAAATAATATTTACGTTATTGATTTTTTATTTATTTATTGGTAATGTGAGTATGCAATTTTAG
GGTTGAAGAGATGTGGGAAGAGCTGTAGGCTAAGATGGACTAATTATCTGCGCCCTAATTTGAAGCATGAG
AGCTTCACTCAACAAGAAGAGGAGATGATTATAACACTTCATGCTACAATCGGAAGCCGGTATTGTTCTCT
CTATCAATTTATTTGATTGAGGATTTTATATCATGATGTTAACTACTGTAGTTTTCATTTTAGTCAATTAG
GAACATTATTTTGTTTGAATAGTTTTAACCTAGAAAGAATTTCTAGTGTAGAGCTGCTAACACAAATATCT
TTACACTGTCCAGATATTAAAGCGGGGCTGAAATAGTAAAAAGGAGGAGGAGGAAAATATCTATAACTATA
TATGACAACCCCAGTTTGAAATTGATTTTAAACATAATTTTATCTAGAAGTGAGATGGACAGGCATCAAAA
ACCTGTACTTTTGAAATGTAGAATGTAAATTAACTCAACAATAGGATATTTATACATTTGAAATTTTCATA
TTTGAAATCTTCTATGTTGAGTTAATCTATGATTATCATCTGGGGTCCACAGAAATTGAAAACATTTGGTT
TTGGAACAGAATTCTAGTTTCAAATGACAGAAAGATCTTTTATAATCTCAAAATGATGAGTAGTTCTATTA
ATTAGTTTTTGCTTACTATTTCAGAAGTTTACCAAAAGACCAGTTTGTTTTGTTGAATTTACATCAAGATG
AGTAGATTGTCTCTTTTGCCAGTTTCCCTACTCATTTTATCATTCACTAATGAGTAATGAGTAGCTAAATT
GTTTAATATTGCACAATAGGTATAAACTGCTAATTTCATGGGTAAATTACACTAAACCCCCATAAACTTTC
AGTGCTTTGTAGTAAACACAGTAAACTTTCAAAATCACACTAAAATCCCAATAACTTTTGCAGAGAACTTC
TATGAACTTCTATGGTAAATTATACTAAACCCTCATGAACTTTCAAAATACCTTTTTATGTTATTTTTTTG
TTCTAATTTTGGGGATTATCTACAAAACTCAGAAAAATCATGGGCGCTCTTTGCAATTCCAAAAAGTTCAT
TGGGGTTTAGTGTTATTTTGAAAATTCATTGGGATATATTGCAAAACTCTAAAAGTTCAAGGGTTTATTGT
AATTTACCCTAATTTAATATTGCACATTATTGTCCCCTTTTGAATGAAATGGGCTAATTAAGGCTTATATT
TCCTTTCGTTATCATGAGATTTGAAAGAACTATTGGTGTAAAGGAAGCTACGGTAGCTAAAAGTTTGGATT
AGTTAAAGAGTCATCTAATTACCTTAGAATTAAAGCTACCGGCATATTTCAATTTCGTGATTGGAGGCAGT
ATGAAAGTGATGATCCCTATCTCCTAACAAATTTAACTAACCATGACGTACACACGATCACTCCCCCTCAC
TCTCTCCAACAGTCACAATTATATTCCTTTTTAGTACTTAATTATGTACATAACACCATGATTATTTTTAG
TACAGGTGGTCGGTAATCGCACATCACCTCCCGGGTCGAACAGACAACGACATAAAGAACCACTGGAACAC
AAAACTGAGCAAAAAACTGTGCCAGCAAGGCATCGACCCCGTCACCCACAAACCCATCTCTCAAATCAAGG
AAACCATCACCACTCTAGCCGCCGCCGCCGCCGCCAACCACCACCTCCTAATCCACCCTCCACCCTTCAAC
ACCCGCGTCAACAGCTGCCTGAGCCGCGACCTCAAGAACGTCCTCCTCTCCAAACCGCAACAATTCTATGA
ACCAACAACAGCCACAAGCACCACATTGGATGAGGTTTATAAGCAGGATAAGGAGATCAAATGGAGCGATT
ATCTCGTCGACGATGTTTTCGTGCCGAACCAAGAGAAGGAATTGGTGGTGAATGGATATGGGAAGGAGAAG
GTGACAAGTGCAGTGGATGAGGAGGTGAGTAGTACTGTGTTTGGAGGTGAAGGGAGTAGTAGCTCGAGTTC
TTTTGTGGAGGGAATATTAGATCAGGGGAGGGAGATGATGATGGAGTTCCCTGAGTTTTTTTATGATTTGC
TGTAGGCGTTTGTGCGTTGAGGATCTTAGTTAGGAACTTCGTGTGGAGTATTAGTAAATTATTTTAAACTT
AGGTTGATGTTTAGTCAGGGTGGTAGTTTCTAGTTATTGTTAGTGGGTGTGGTCATGTGATATCTTCTTTT
AACTGACAAGGGCTTACTTAAATAATTGGATTGTCTACTTCAAAACTAGCTAATTATGTCATCTAGTTATT
GCCATTTTTCTGAATAAAATGAATTTTAAGAGAGAAATCATAACAAG

Figure S6. The sequence of the genomic region of *AoMYB35*. The regions corresponding to the 5’ and 3’ untranslated regions of *AoMYB35* mRNA are indicated as light grey, and the regions corresponding to the coding sequence are indicated as dark grey. The boxed sequence is the putative TATA box.

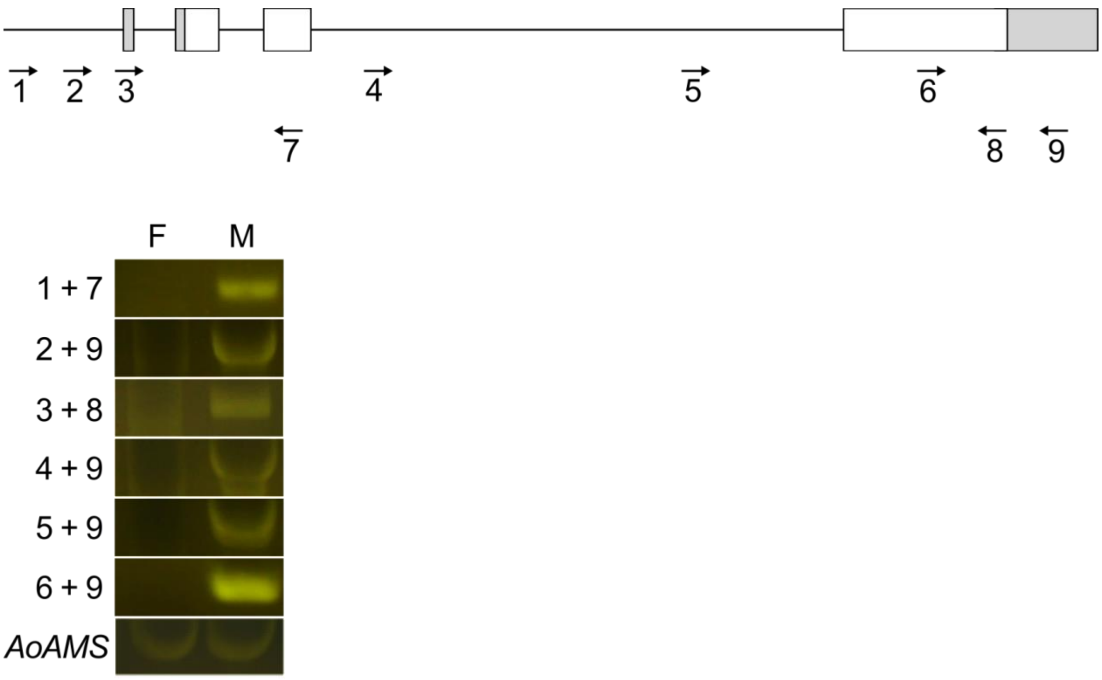

Figure S7. Genomic PCR analysis of *AoMYB35* with various primers. In the upper panel, the untranslated regions and the protein-coding region of *AoMYB35* are shown as grey boxes and white boxes, respectively, and annealing sites of the primers 1-9 are indicated. Genomic DNA was extracted from female (F) and male (M) plants of the *Asparagus officinalis* cultivar NJ264, and used as PCR templates. In the lower panel, primer combinations are indicated, and the signals of *AoAMS* are shown as a control. Experiments were performed three times for each primer combination, and representative results are shown.

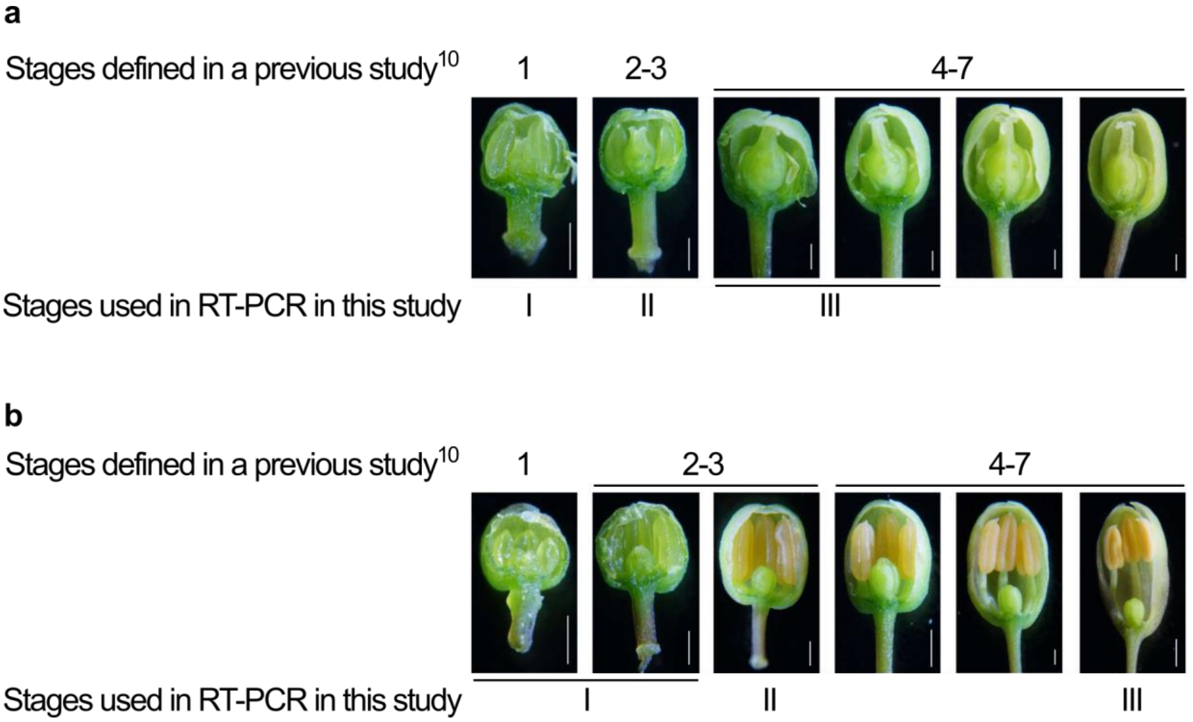

Figure S8. Stages of flower development in *Asparagus officinalis*. For both female (panel a) and male flowers (b), the classification of the stages 1-7 in the previous study[10] and the classification of the stages I-III in this study are both based on the extent of growth of the pistil and stamens. Bars = 1 mm.

a

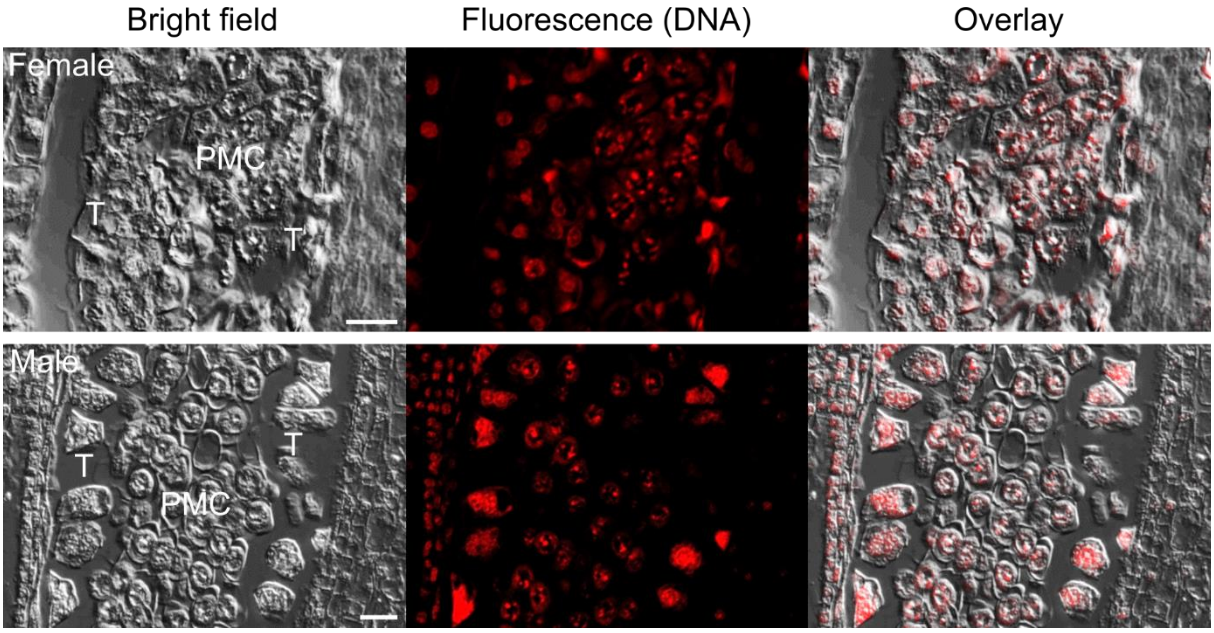
Bright field
Fluorescence (DNA)
Overlay
Female
PMC
T
T
Male
T
T
PMC

b

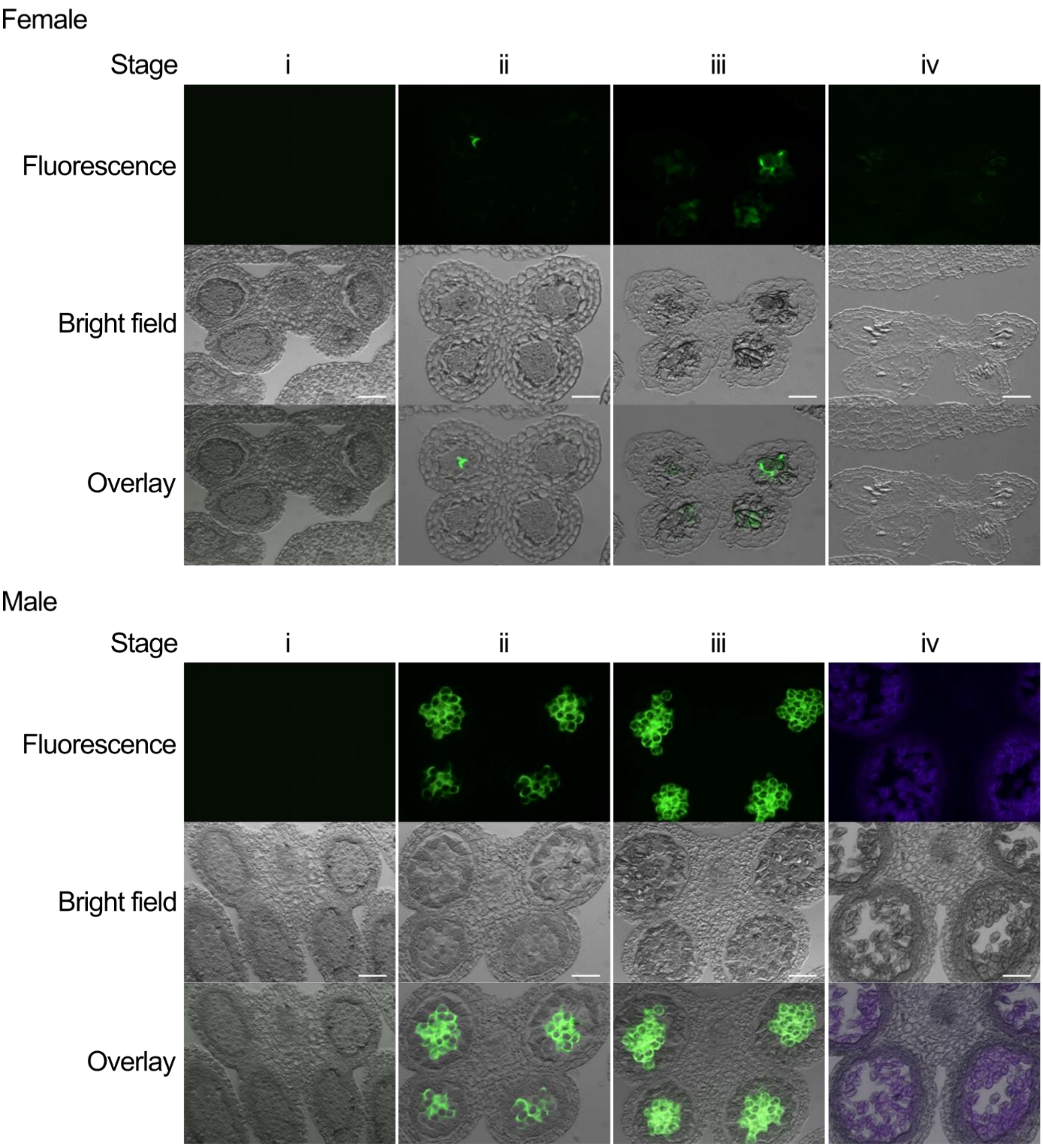
Female
Stage
i
ii
iii
iv
Fluorescence
Bright field
Overlay
Male
Stage
i
ii
iii
iv
Fluorescence
Bright field
Overlay

Figure S9. DNA condensation and callose deposition in anthers of *Asparagus officinalis*. (a) DNA in cells in anthers in female and male flowers was detected with Feulgen stain. Experiments were repeated more than three times, and representative images are shown. T and PMC indicate the positions of tapetal cells and pollen mother cells, respectively. Bars = 20 μm. (b) Callose in anthers in female and male flowers was detected with aniline blue, and is shown in green. Autofluorescence of pollen grains is shown in purple. The stage i corresponds to the premeiotic stage for both female and male flowers. In the stage ii female flowers, both tapetal cells and pollen mother cells are still present. In the stage ii male flowers, callose is deposited around pollen mother cells. In the stage iii female flowers, pollen mother cells are still present, but tapetal cells are lost. In the stage iii male flowers, pollen tetrads are present. In the stage iv female flowers, pollen mother cells are deformed and shrunken. In the stage iv male flowers, pollen grains are present and the deposited callose is lost. Experiments were repeated three times for each stage for both female and male flowers, and representative images are shown. Bars = 100 μm.

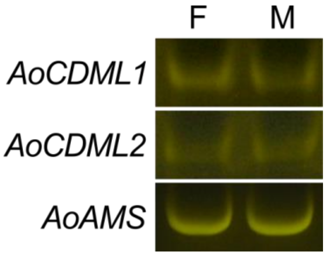

Figure S10. Genomic PCR analysis of *AoCDML1* and *AoCDML2*. Genomic DNA was prepared from female (F) and male (M) plants of the *Asparagus officinalis* cultivar NJ264, and used as the template. Experiments were repeated three times, and representative images are shown.
